# Refractive errors in patients with newly diagnosed diabetes mellitus

**DOI:** 10.12669/pjms.316.8204

**Published:** 2015

**Authors:** Abdülhekim Yarbağ, Hayrullah Yazar, Mehmet Akdoğan, Ahmet Pekgör, Suleyman Kaleli

**Affiliations:** 1 Dr. Abdülhekim Yarbağ, SMECC. Abdulhekim Yarbag, Special Marmara Eye Clinic Center, Sakarya, Turkey; 2 Dr. Hayrullah Yazar, SAUFM. Assistant Professor, Sakarya University Faculty of Medicine, Sakarya, Turkey; 3 Dr. Mehmet Akdoğan, SAUFM. Professor, Sakarya University Faculty of Medicine, Sakarya, Turkey; 4 Ahmet Pekgör, NEUSF. Necmettin Erbakan University Department of Statistics Faculty of Sciences; 5 Dr. Suleyman Kaleli, SAUFM. Associate Professor, Sakarya University Faculty of Medicine, Sakarya, Turkey

**Keywords:** Diabetes mellitus, Fasting plasma glucose, Hyperopia, Refractive error

## Abstract

**Background and Objective:**

Diabetes mellitus is a complex metabolic disorder that involves the small blood vessels, often causing widespread damage to tissues, including the eyes’ optic refractive error. In patients with newly diagnosed diabetes mellitus who have unstable blood glucose levels, refraction may be incorrect. We aimed to investigate refraction in patients who were recently diagnosed with diabetes and treated at our centre.

**Methods:**

This prospective study was performed from February 2013 to January 2014. Patients were diagnosed with diabetes mellitus using laboratory biochemical tests and clinical examination. Venous fasting plasma glucose (fpg) levels were measured along with refractive errors. Two measurements were taken: initially and after four weeks. The last difference between the initial and end refractive measurements were evaluated.

**Results:**

Our patients were 100 males and 30 females who had been newly diagnosed with type II DM. The refractive and fpg levels were measured twice in all patients. The average values of the initial measurements were as follows: fpg level, 415 mg/dl; average refractive value, +2.5 D (Dioptres). The average end of period measurements were fpg, 203 mg/dl; average refractive value, +0.75 D. There is a statistically significant difference between after four weeks measurements with initially measurements of fasting plasma glucose (fpg) levels (p<0.05) and there is a statistically significant relationship between changes in fpg changes with glasses ID (p<0.05) and the disappearance of blurred vision (to be greater than 50% success rate) were statistically significant (p<0.05). Also, were detected upon all these results the absence of any age and sex effects (p>0.05).

**Conclusions:**

Refractive error is affected in patients with newly diagnosed diabetes mellitus; therefore, plasma glucose levels should be considered in the selection of glasses.

## INTRODUCTION

Although the benefits of aggressive glucose control on refractive error in type 2 diabetes mellitus patients are well established, management of the effects can be complicated, particularly due to difficult glycaemic control in new cases. Therefore, we will discuss in some detail the process of diabetes mellitus diagnosis and optics refraction.

Diabetes mellitus is a complex metabolic disorder that involves the small blood vessels, often causing widespread damage to tissues, including the eyes, kidneys (end stage renal disease; ESRD), etc.^1^,^2^ Ocular complications arise approximately 20 years after onset despite apparently adequate diabetic control. Improved treatment measures (e.g., improved insulin, antibiotics) that have lengthened the life span of diabetics have actually resulted in a marked increase in the incidence of retinopathy and other ocular complications. The possibility of diabetes should be considered in all patients with unexplained retinopathy, cataracts, extra-ocular muscle palsy, optic neuropathy, or sudden changes in refractive error.

Absence of glycosuria or a normal fasting plasma glucose (fpg) level does not rule out a diagnosis of diabetes.^3^ In the post-absorptive state, the concentration of plasma glucose in healthy individuals is within the range 4.5-5.5 mmol/L (65-95 mg/dL). After the ingestion of a carbohydrate meal, it may rise to 6.5-7.2 mmol/L. During fasting, the levels fall to around 3.3-3.9 mmol/L.^4^ A sudden decrease in blood glucose will cause seizures, as in insulin overdose, owing to the immediate dependence on glucose of the brain. However, much lower concentrations can be tolerated, provided adaptation is progressive; e.g., rats fed a high-fat diet behave normally with a plasma glucose concentration as low as 1.1 mmol/L.^4^

Glycosuria is not seen in healthy individuals, but when the blood glucose rises to relatively high levels, the kidney exerts a regulatory effect. Glucose is continuously filtered by the glomeruli but is ordinarily returned completely to the blood by the absorptive system of the renal tubules. Glycosuria of renal origin may result either from inherited defects in the kidney, or it may be acquired as a result of disease processes. The presence of glycosuria is frequently an indication of diabetes mellitus. The capacity of the tubular system to reabsorb glucose is limited to a rate of about 350 mg/min. When the blood glucose level is elevated, the glomerular filtrate may contain more glucose than can be absorbed; the excess is then excreted into the urine. In normal individuals, glycosuria occurs when the venous plasma glucose concentration exceeds 9.5-10.0 mmol/L. This is termed the renal glucose threshold.^4^

Optics refraction, the correct interpretation of visual information, depends on the eye’s ability to focus incoming rays of light onto the retina. An understanding of this process and how it is influenced by normal variations or ocular disease is essential to the successful use of any optical aid; e.g. corrective lenses, contact lenses, intraocular lenses, or low-vision aids. Good diabetic control retards the development of retinopathy and other diabetic complications such as increased refractive error.^5^

The diabetic state causes sudden changes in the refraction of the lens, especially when diabetes is not well controlled. Changes in blood glucose levels alter the refractive power by as much as 3 or 4 dioptres of hyperopia or myopia. This results in blurred vision. Such changes do not occur when good glycaemic control is achieved. The blood glucose concentration is regulated within narrow limits in healthy individuals, but these limits are disrupted in patients with diabetes.^3^-^5^

## METHODS

The present study included 130 adult patients with a new diagnosis of diabetes mellitus. The study was performed after the approval [By The Ethics Committee, Sakarya University Medicine Faculty, Number: 71522473/050.01./69] from February 2013- January 2014 in the Marmara Eye Center, and the following criteria were adopted. The age and sex of the patients were recorded. All patients had been newly diagnosed with diabetes mellitus using laboratory biochemical tests and clinical examination.

Each patient was monitored for 4 weeks. Measurements were taken at the beginning and end of this period. Measurements and evaluation e had been made simultaneously as fpg levels and refractive error. Refractive errors of the patients were measured twice, initially at a high fbg and second at a low one. The difference between the first and second refractive measurements was evaluated. Additionally, venous fasting plasma glucose levels with diopter changes evaluated a statistically association. Inclusion criteria were newly diagnosed diabetes mellitus adult patients who did not have retinopathy. All patients were tested for blurred vision. The statistical significance of differences between complications, sex, age, transmission paths, guideline mean values, and our study values were evaluated (P< 0.05). The patients were statistically evaluated and for all statistical analysis was used (Paired Samplest Test, Spearman Correlation, Kolmogorov Smirnov Normality Test, Binomial Test. For all analysis, p values <0.05 were considered significant. The Statistical Package for the Social Sciences version 21.0 (IBM SPSS).

## RESULTS

Our patients, 100 males and 30 females, had been newly diagnosed with type II DM. The refraction values and fpg levels were measured twice in all patients. The average values of the initial measurements were as follows: fpg level, 415 mg/dl; average refractive value, +2.5 D (Dioptres). The average end-of-period measurements were fpg, 203 mg/dl; average refractive value, +0.75 D. The refractive values were different between the two measurements (Table-I). There is a statistically significant difference between after four weeks measurements with initially measurements (p<0.05) (paired samples t test).

**Table-I:** Comparison of fpg levels and refractive error and change.

The process	The measurements		The change (%)
	Initially (Mean)	After four weeks (Mean)	
FPG level	415mg/dl	203mg/dl	51.08
Refractive Error	2.5D	0.75D	60
Blurred vision	130 patients	0	100

***Note:*** There is a statistically significant difference between after four weeks measurements with initially measurements of fasting plasma glucose (fpg) levels (p<0.05) and there is a statistically significant relationship between changes in fpg changes with glasses ID (p<0.05) and the disappearance of blurred vision (to be greater than 50% success rate) were statistically significant (p<0.05).

The changes were identified; end-of-period measurements average fpg levels, 51.08%; average refractive values, 60% (Table-I). These change (end-of-period) were statistically significant (p<0.05) (spearman correlation). Also were detected upon all these results, the absence of any age and sex effects (p>0.05). Another noteworthy finding is the detection of blurred vision in all the patients in the first condition that was eliminated with the use of corrective lenses after 4 weeks. The disappearance of blurred vision (to be greater than 50% success rate) were statistically significant (p<0.05).

## DISCUSSION

Hyperglycemia is the major cause of transient refractive changes in diabetic patients. In the 19th century it was recognised that the vision of diabetic patients is influenced by their changing blood glucose concentrations.^6^ In 1925 Dyke et al. refracted the eyes of two patients with diabetic ketoacidosis and again after their blood glucose had returned to the normal range. He concluded that hyperglycemia produced myopia and that lowering the blood sugar resulted in hyperopia. Since that time, no systematic studies to further define the relationship of serum glucose to changes in refraction have been performed. However, many differing opinions regarding this relationship are expressed.^7^ Our study was the first to re-examine this phenomenon.

Grant et al. suggested that the change in vision that accompanied chronic changes in serum glucose paralleled the degree of change in glucose concentration.^6^-^8^ He also. suggested that the change in vision that accompanied chronic changes in serum glucose paralleled the degree of change in glucose concentration. These inferences agrees with our findings (Table-I). Their studies suggest that an increase in serum glucose concentration, whether acute or chronic, is invariably associated with a more myopic or less hyperopic change in vision as long as the crystalline lens of the eye is intact. Additionally, they found that such changes do not occur in an eye from which the lens has been removed.

Kristian et al. investigated the effect of intensive glycemic control on hyperglycemia-induced temporary changes in refractive error in Type 1 diabetic patients without retinopathy. Their study assessed the effect of variations in blood glucose level on the multifocal electroretinogram.^9^ Their study design was similar to ours in regard to the presence of retinopathy, although it differed in terms of the type of diabetes. However, in their studies with our study is parallel to investigate changes in blood sugar levels.^9^ That is; they compared hyperglycemia associated with an overall decrease in the implicit times and an increase in blood glucose levels.

Bozkurt et al. also investigated the effect of glycaemic control on refractive changes in diabetic patients with hyperglycemia.^10^ They measured blood glucose repeatedly over the same period and their results demonstrated the mechanisms by which elevated glucose affects cellular metabolism with a time course consistent with the transient nature of the effect observed. In our study, the fasting venous blood glucose was analysed in newly diagnosed diabetes patients.

In a study conducted by Elisabeth et al. in patients with controlled diabetes, refractive and visual acuity test results were highly reproducible and stable in patients with variable blood glucose levels under routine care. They found that refraction was completely stable in 43 of the 53 patients. They also found no significant difference in blood glucose variability among the diabetic patients.^11^ Their results suggest that refractive changes are minimal and assessments are highly reproducible provided that a standard method is used for testing, within a wide range of blood glucose levels in diabetic patients under routine care with different degrees of retinopathy.^11^ However, in our study, refractive error was altered markedly by fasting plasma glucose levels. This difference could have been due to the fact that our patients had been newly diagnosed with diabetes.

Tatsuyuki et al. studied blurred vision in diabetes mellitus patients.^12^ Although the accurate incidence of transitory refractory change in diabetic patients is unknown, it ranges from 5 to 50% in untreated or uncontrolled patients. However, Bradley et al. reported that working showed higher incidence of 90%, which agrees with the recent observation.^13^ Moreover, their results indicated that the magnitude of fpg decrement during the treatment was closely associated with the fluctuation of refraction of the eyes in diabetic patients, as reported by Holton.^14^,^15^ Our study results showed blurred vision in 100% of patients.

## CONCLUSIONS

In diabetic patients refraction is affected by blood glucose; therefore, monitoring of this value is important in the selection of corrective lenses. We recommend that eyeglasses should not be prescribed for 4 weeks in patients who are newly diagnosed with diabetes mellitus.
